# Perception, attitude, and practice toward research among medical students in Hadhramout University, Yemen

**DOI:** 10.1186/s12909-023-04287-0

**Published:** 2023-11-10

**Authors:** Abdullah Bin-Ghouth, Suha Ali Batarfi, AbdulRahman Hashim Abonemi, Ahmed Sadeq Maknoon, Ahmad Sa’ad Alkhanbshi, AlwiAbobaker Khred, Amal Abdullah Bawazir, Areej Abdullah Ba-Jaber, Aiman Abdullah Rezq, Isra’aAlwi Maknoon, Khadijah Ahmed Badheeb, Maha Salah Alkathiri, Majedah Ahmed Ba-Rbaa, Mustafa Dhaiban, Omar Ali Bagumaish, Omar Saleh Baslasel, Roua’a Abdullah Ba-rady, Souha Algadry, Tagwa Omar Bazanboor

**Affiliations:** 1 Department of Community Medicine, Hadharamout University College of Medicine (HUCOM), Hadhramout, Yemen; 2 Community Medicine, Department of Community Medicine, Hadharamout University College of Medicine (HUCOM), Hadhramout, Yemen; 3 Hadharamout University College of Medicine (HUCOM), Hadhramout, Yemen; 4 Department of Community Medicine, Hadharamout University College of Medicine (HUCOM), Hadhramout University, 8892, Mukalla, Fwah, Yemen

**Keywords:** Medical students, Undergraduate research, Yemen

## Abstract

**Background:**

Research is an important element in the improvement of the quality of health services provided to the public. It is documented that globally; medical students apply research in their school life. In Hadhramaut University, medical students work on research in groups, and it is an important part of the curriculum. There is a formal assessment of the student’s research, but there is still a gap regarding individual viewpoints and challenges faced. This study aimed to assess perception, attitude, and practice toward research among medical students at Hadhramout University.

**Methods:**

This is a cross-sectional descriptive study which was conducted among medical students. This study was undertaken in Hadhramout University in Al-Mukalla district, Yemen, during the academic year 2016–2017. A self-administered pilot-tested questionnaire was used for data collection to assess perception, attitude, and practice toward the research during the educational year 2016–2017.

**Results:**

A total of 265 completed responses were received. The majority had a low Knowledge score (72%). However, the majority had a positive attitude toward research (90.9%). Eighty-three students reported participation in research work. However, (44.4%) expressed research interest. Many barriers were highlighted by students including a lack of time (78.4%) and a lack of training in statistics (75.9%).

**Conclusion:**

The study identified several barriers for undergraduate medical students to undertake research. It is important that these barriers should be addressed in curriculum development, so that students can retain their motivation to engage effectively in research.

## Background

Research is the generation of new knowledge, science or invention by using scientific methods and skills [1, 2]. Health research training is an important component of medical education to teach critical thinking and reasoning skills [3–5]. The scientific research methodology including topic selection, analysis and statement of the problem and literature review which are highly important processes. It including also formulation of the research objectives, determining study designs, study population and sample size determination, and data collection, processing, and analysis [1, 6].

Health research seeks to answer questions about the health state, disease, or risk factors. It also directs us to the causes of ill health, the effectiveness of prevention and curative intervention, and also has an impact on health care programs policy and services [7, 8]. Moreover, research has the capacity to broaden student’s scientific training and helping them pursue their careers in academic medicine [9].

During the last decade, there has been a renewed emphasis on the medical student research experience. Many programs have been developed in different countries such as those two large United States programs that have sought to engage students during this critical period of training. These programs are the National Institutes of Health (NIH) sponsored Medical Student Research Fellowship Programs (MSRFs) [10] and the Doris Duke Clinical Research Fellowship (CRF) Program [11]. Other examples are those that developed in Europe [12] and specifically in the Netherlands [13].

Many students agree that there would be no progress of humankind without the progress of science. Moreover, the basis of any medical progress is the use of scientific methodology. For example, knowledge of scientific methodology is essential for obtaining accurate objective data so the research is important in the medical field.

However, some students do not agree that research will be a part of the future, and most of them as a result do not present any research [2, 14, 15]. Some of them agreed that some participation in research was likely valuable within their medical education. They showed that research should be made mandatory in the curriculum. Some of the suggestions stated that more time in medical school should be set aside to allow participation in research endeavors. Prompting that this would lead to improved research skills, and reinforce a teamwork spirit and more research publications [2, 14−16].

An important factor underlying any study is the researchers’ beliefs, because it motivates individuals to undertake a study in the first place [17]. The attitude to health research stems from the researchers’ curiosity and interest in a particular subject or their wish to solve a problem within a community [18].

Students had faced many barriers when conducting the research activities. Some of those reported barriers including: no enough time, supervisor support and guidance, inadequate training in research methodology, hard to publish research during medical school [19]. Uncertainty about the ability to complete a study (lack of research self-efficacy), [20] lack of research interest [21] and limited access to data sources (i.e., internet), materials and equipment [22].

In Hadhramaut University, medical students work on research in groups and it is an important part of their program. Students in year two and year three acquire knowledge about epidemiology, research methodology and statistics. They prepare two proposals under supervision of community medicine department; one proposal for public health services, and another proposal is for observational clinical research of common diseases presented in the hospitals. There is a formal assessment of the student’s research. However, there is a disparity regarding individual viewpoints and many barriers still exists causing persistent challenges.

To our knowledge, there had been no previous published research that addressed these issues in Yemen. Our study aims to assess perception, attitude, and practice toward research among medical students in Hadhramout University College of Medicine (HUCOM) during the academic year 2016–2017.

## Methods

### Study design

Cross-sectional study undertaken among the last three clinical levels of medical students education (year 4, 5 and 6) of Hadramout University in Yemen.

### Setting of the study

The study was conducted in Hadramout University College of Medicine (HUCOM). HUCOM is one of the seven colleges of Hadramout University at eastern Yemen. It was established in September 1997. A problem-based learning (PBL) curriculum is taught with a community oriented medical education approach.

### Study subjects

418 students enrolled in the study, 154 students were from the fourth year, 134 students were from the fifth year, and 130 students were from the sixth year.

### Data collection

The data was collected by using a self-administered pre-tested questionnaire. The questionnaire was designed and validated by experts (professors of community medicine) from different universities in the Arab world. The survey questions were collected from previous studies [14, 17, 18, 19] covering knowledge, attitudes and practice of medical students toward research and the barriers they faced. The questions were modified and developed to fit our objectives and to assess knowledge, attitudes and participatory role of medical students in medical research.

The questionnaire was distributed to ten academic staff from eight universities in Yemen, Iraq, Saudi Arabia and Egypt. The approach we used to collect inputs of the experts was a form using three columns for their input: the language, scientific content and any comments, and corrections were done before and after we conduct the pre-test. Questions are well understandable and the corrections applied were around the language. Pre-test was conducted with 30 students. Reliability test Cronbach’s Alpha was 0.79.

**The questionnaire**: The questionnaire was divided into seven sections as follow: (1) Personal data (Age, Gender, Academic level, Name not included), (2) Student’s perception toward research, (3) Student’s attitude toward research, (4) Students practice toward research, (5) Barriers toward research, (6) Relevance of the undergraduates’ research, (7) Suggestions to improve engagement of students in research.

#### Knowledge and attitude score

The knowledge score consisted of six items. A score of 1 is given for each correct answer, while zero for the wrong answer. The total knowledge score ranged from zero to six and then was classified as High knowledge score (above or equal to the mean), and low knowledge (if it was below the mean). Attitudes towards research were assessed by eight questions. The answers were measured by a 5-point Likert scale ranging from strongly disagree (score 1) to strongly agree (score 5). So, the range of the total attitude score was ranged from 0 to 40, then it was classified as Positive attitude (above or equal to the mean), and negative attitude (if it was below the mean).

### Data analysis

The collected data was checked, coded, entered, and analyzed using a statistical package for social science (SPSS v20). The descriptive statistical tools used as appropriate were frequencies, mean and standard deviation. The outcome variables were student knowledge, attitude and practice, while gender and the academic level were the main independent variables. The statistical tests used for analysis were one-way analysis of variance (ANOVA) for multiple comparisons, and after checking normality we used t-test for continuous variables. The criteria of significance were considered at a P-value of 0.05.

## Results

### The response rate

Out of 418 questionnaires distributed, 265 students respond and returned completed questionnaires giving the response rate of 66.5%. (104 fourth-year students, 86 fifth-year students, 75 sixth-year students).

### Participants characteristics

The mean age of participants was 23.9 years (± 1.5). The majority of students were in the age group 23–26 years, 134 of students (50.6%) were males and (49.4%) were females (Table 1).

**Table 1:** The demographical characteristic of the participant

Characteristics		Frequency	Percentage
Age group (Mean age (23.9 ± 1.5)	20–22	37	14.2%
	23–25	191	73.5%
	26–29	32	12.3%
Sex	Male	134	50.6
	Female	131	49.4
Academic year	6 year	75	28.3
	5 year	86	32.5
	4 year	104	39.2

#### The student’s knowledge about having research

**Fig. 1:**
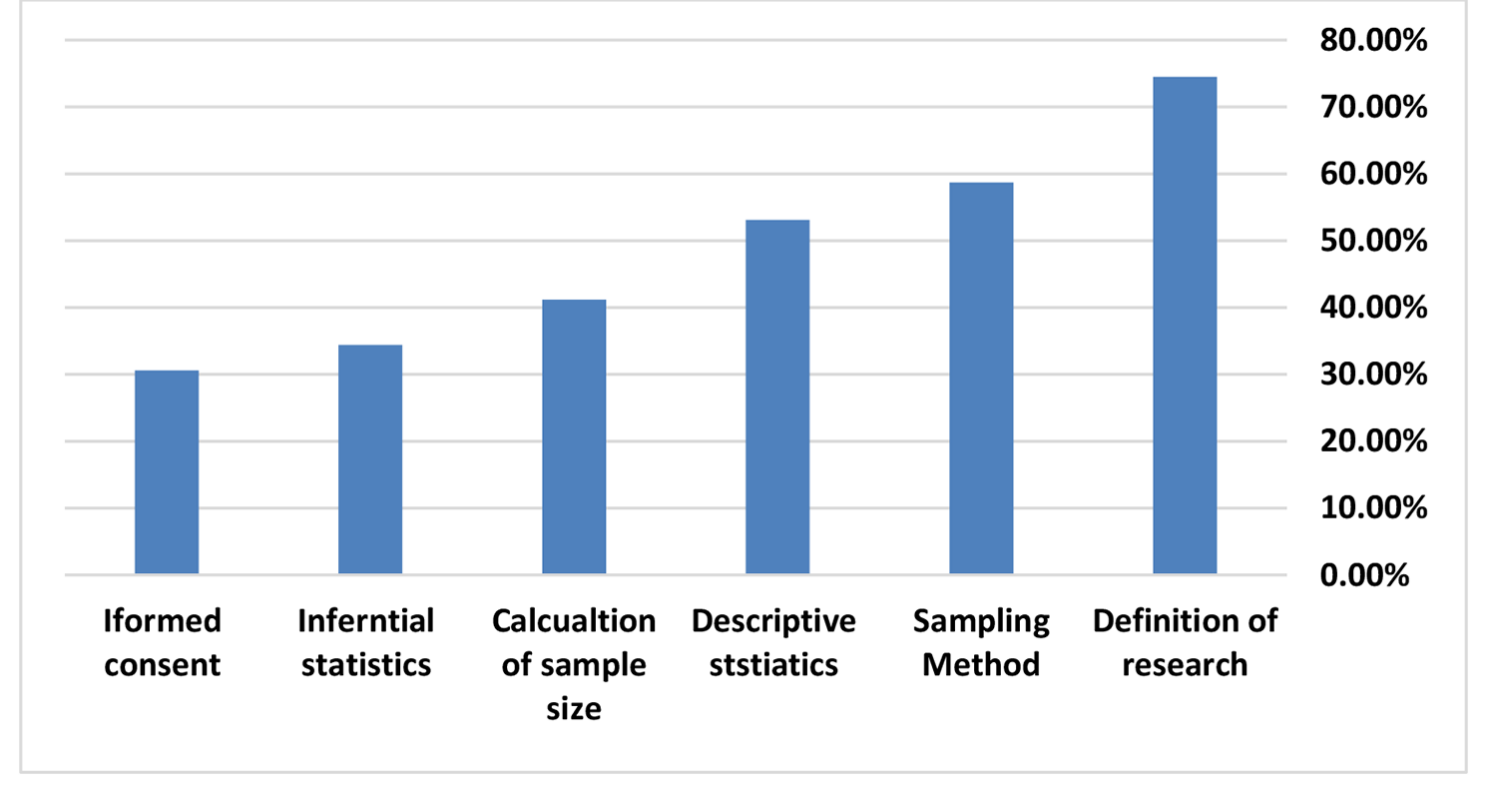
The student’s knowledge about research

The highest percentage of the students (74.5%) knew about the research concepts and they knew the importance of a representative sample (58.7%), but only 23.3% of students understand the role of sample size in the generalize ability of the obtained results. About 53% of the students were able to define descriptive statistics, in contrast to inferential statistics where only 34.4% of students were able to define. Regarding research ethics, only 30.6% were able to identify the correct definition of the informed consent Figure 1.

#### The student’s attitudes toward research

About 41.8% of students strongly agreed that engaging medical students in research was important. And about 37.4% of students is strongly agreed that studying research methodology course is important. Additionally, 38.3% agreed that understanding research increases the burden of the overloaded curriculum on medical students. When asked about whether medical students can plan and conduct research without supervision, 33.2% of students disagreed, and only 5.8% strongly agreed. While 30.4% of students agreed that research participation should be compulsory to all medical students and 39.3% is also agreed that understanding statistics is important specially to analyze and interpret data. About 30.7% of students agreed that research is interesting and 38.9% reported that they have learned most when undertaking research (Table 2).

**Table 2:** The student’sattitude toward research

Items	Strongly agree n (%)	Agree n (%)	Not determined n (%)	Disagree n (%)	Strongly disagree n (%)
Engaging medical students in research is important	110(41.8)	90 (34.2)	31 (11.8)	25 (9.5)	7 (2.7)
Studying research methodology course is important	98(37.4)	96(36.6)	42(16)	17(6.5)	9(3.4)
Understanding research increases the burden of the overloaded curriculum on medical student	76(29.1)	100(38.3)	47(18)	24(9.2)	14(5.4)
Medical students can plan, conduct research without supervision	15(5.8)	35(13.5)	34(20.8)	86(33.2)	69(26.6)
Research participation should be compulsory to all medical students.	48(18.7)	78(30.4)	67(26.1)	39(13.2)	25(9.7)
Understanding statistics is important for me to analyze and interpret data	93(36.2)	101(39.3)	39(12.8)	20(7.8)	10(3.9)
I am very interested in research	35(13.5)	80(30.7)	59(22.6)	45(17.2)	42(16.1)
I have learned most when undertaking my research	57(21.8)	102(38.9)	49(18.7)	27(10.3)	27(10.3)

Regarding the distribution of medical students in Hadhramout University on the Knowledge and attitude Scale, the overall mean scores of students on knowledge and attitude were 2.72 (± 1.22) and 27.66 (± 5.31) respectively. The majority of students had low Knowledge scores (72%). However, the majority of them had a positive attitude toward research (90.9%) (Table 3).

**Table 3:** Knowledge and attitude score

Item		Frequency	Percent
Knowledge	High	73	28.0%
	Low	188	72.0%
(mean ± SD)	2.72 ± 1.22		
Attitude	Positive	240	90.9%
	Negative	24	9.1%
(mean ± SD)	27.66 ± 5.31		

The present study found that there was a significant difference between sex in terms of knowledge score as female students (2.99 ± 1.24) having greater knowledge than males (2.45 ± 1.16) (P-value 0.000), but there was no significant difference between sex regarding attitude sore (P-value 0.064) (Table 4).

**Table 4:** Gender variation in Knowledge and attitude score regarding Research among medical students

Knowledge and attitude	Male	Female	T-value	P-value
Knowledge score mean (SD )*	2.45 ± 1.16	2.99 ± 1.24	-3.613	0.000**
Attitude score mean (SD )	27.06 ± 5.55	28.27 ± 5.01	-1.860	0.064

*SD = Standard Deviation

**Significant at p-value < 0.05

There was no significant difference regarding knowledge of and attitude to research concerning academic years (as p-value 0.243 and 0.719 respectively) (Table 5).

**Table 5:** Analysis of variance of knowledge and attitude score regarding research among medical studentsby academic years

Item	Academic years			T value	P-value
	Fourth-year	Fifth-year	Sixth year		
Knowledge score mean (SD)	2.87 ± 1.09	2.65 ± 1.21	2.57 ± 1.40	1.422	0.243
Attitude score mean (SD )	27.62 ± 5.56	27.37 ± 4.96	28.05 ± 5.39	0.330	0.719

#### The student’s practice toward the research process

(48.3%) of students were frequently participated in developing a research proposal, (45%) sometimes conducting research work, (57%) frequently participated in data collection, (39.2%) frequently participated in data entry, (37.2%) participated in statistical analysis, and (41.2%) sometimes participated in writing the research final report, (42.2%) never presented a research paper in a conference or college meeting, (56.4%) never participated in publishing a research paper in a journal, (39.8%) sometimes calculated the sample size, (40.1%) never designed tables and graphs correctly (Table 6).

**Table 6:** The student’s practice toward research

Items	Frequently n (%)	Sometimes n (%)	Never n (%)
I participated in developing a research proposal	127(48.3)	114(43.3)	22(8.4)
I participated in conducting research work (in general)	99(38.1)	117(45)	44(16.9)
I participated in the data collection stage	149(57)	79(30.9)	31(12.1)
I participated in data entry using a statistical software program	102(39.2)	79(30.4)	79(30.4)
I participated in the statistical analysis of the obtained data	98(37.5)	97(37.2)	66(25.3)
I participated in writing the research final report	82(31.3)	108(41.2)	72(27.5)
I presented a research paper at a conference or my college meeting	63(24.4)	86(33.3)	109(42.2)
I participated in publishing a research paper in a journal	43(16.7)	69(26.8)	145(56.4)
I calculated the sample size correctly	87(33.3)	104(39.8)	70(26.8)
I designed tables and graphs correctly	61(23.3)	96(36.6)	105(40.1)

**Fig. 2:**
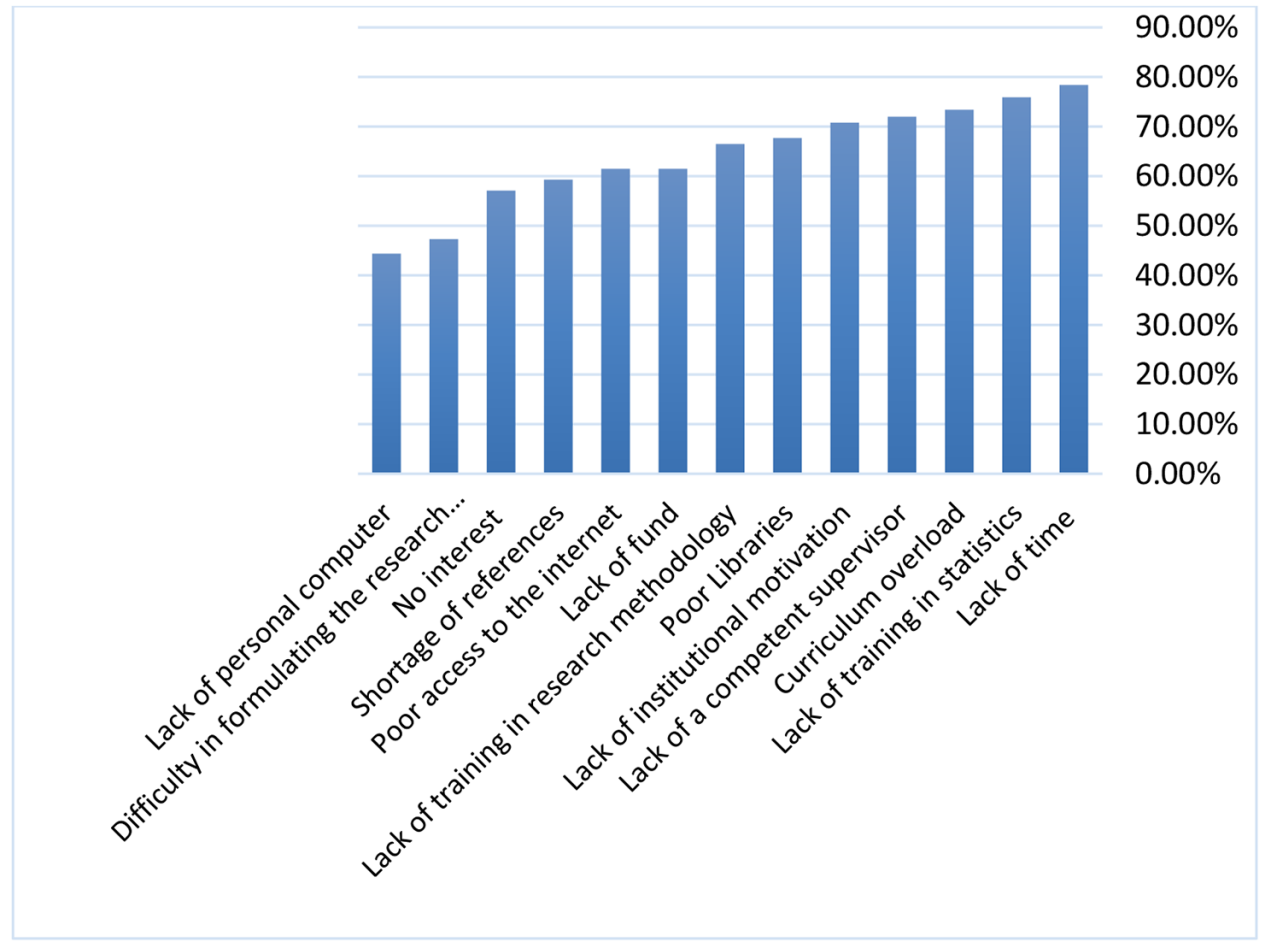
The student’s barriers to research (N = 265)

The greatest barriers we found to conduct research were: firstly most students (78.4%) stated that lack of time is the main barrier for conducting research. Only 24.6% of the participants agreed that there was sufficient time available. Secondly, a lack of statistical knowledge (75.9%) and thirdly, overloaded curricula (73.4%). In addition, 72% of the students reported that there was a lack of competent supervisors. Other barriers included insufficient training in research methodology (66.5%), poor access to the internet (61.5%) and lack of interest in research (57.1%) Figure 2.

### The student’s relevance of the undergraduates’ research

**Fig. 3:**
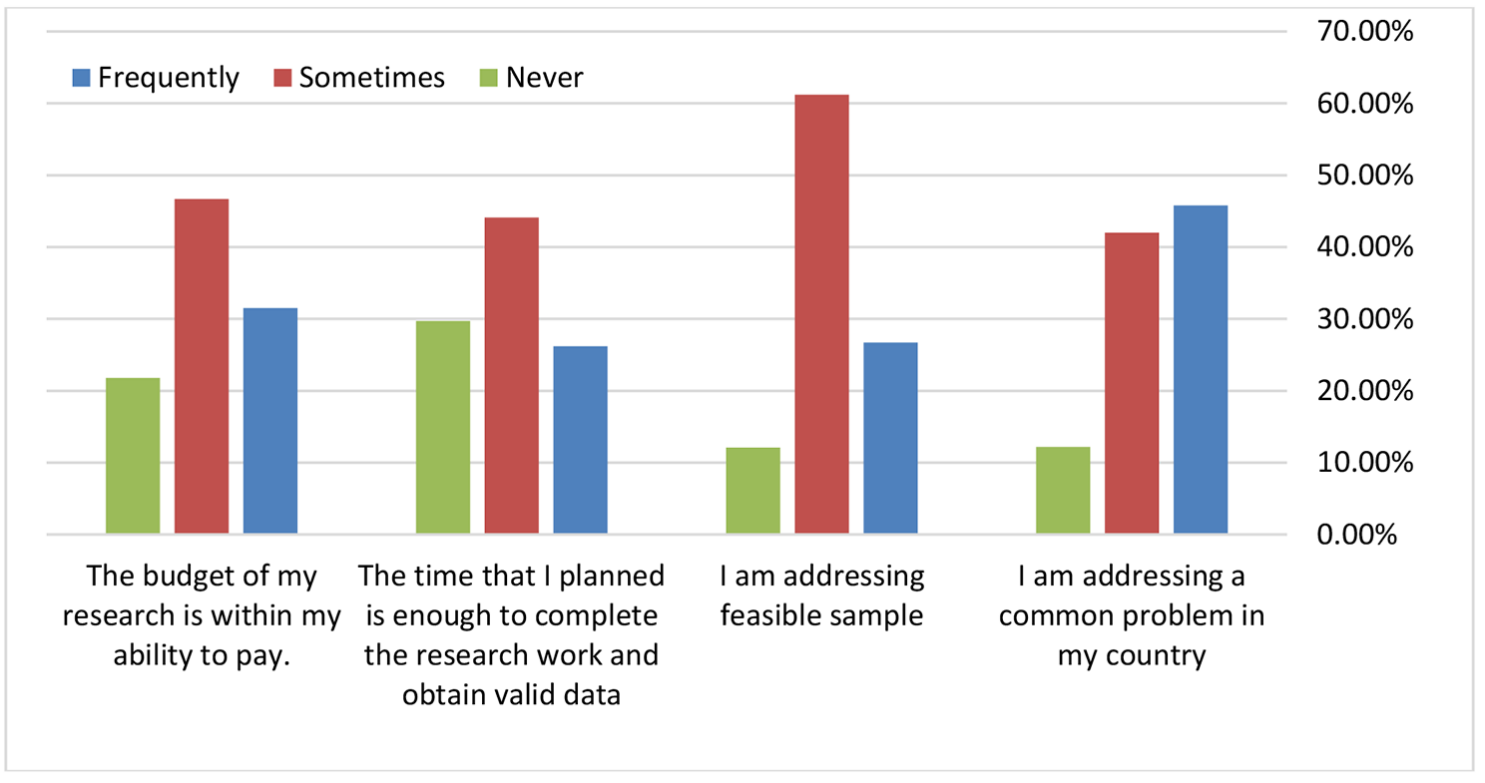
The student’s relevance of the undergraduates ’research

About 45.8% of students frequently addressed a common problem in their country through research. A high percentage (61.2%) of students sometimes addressed a research question with a feasible sample, (44.1%) of students sometimes had enough time to complete research, (46.7%) of students sometimes had a budget to pay for their research, (43.5%) of students had never published their research in journals Figure 3.

### The student’s suggestions to improve engagement of undergraduates in research

**Fig. 4:**
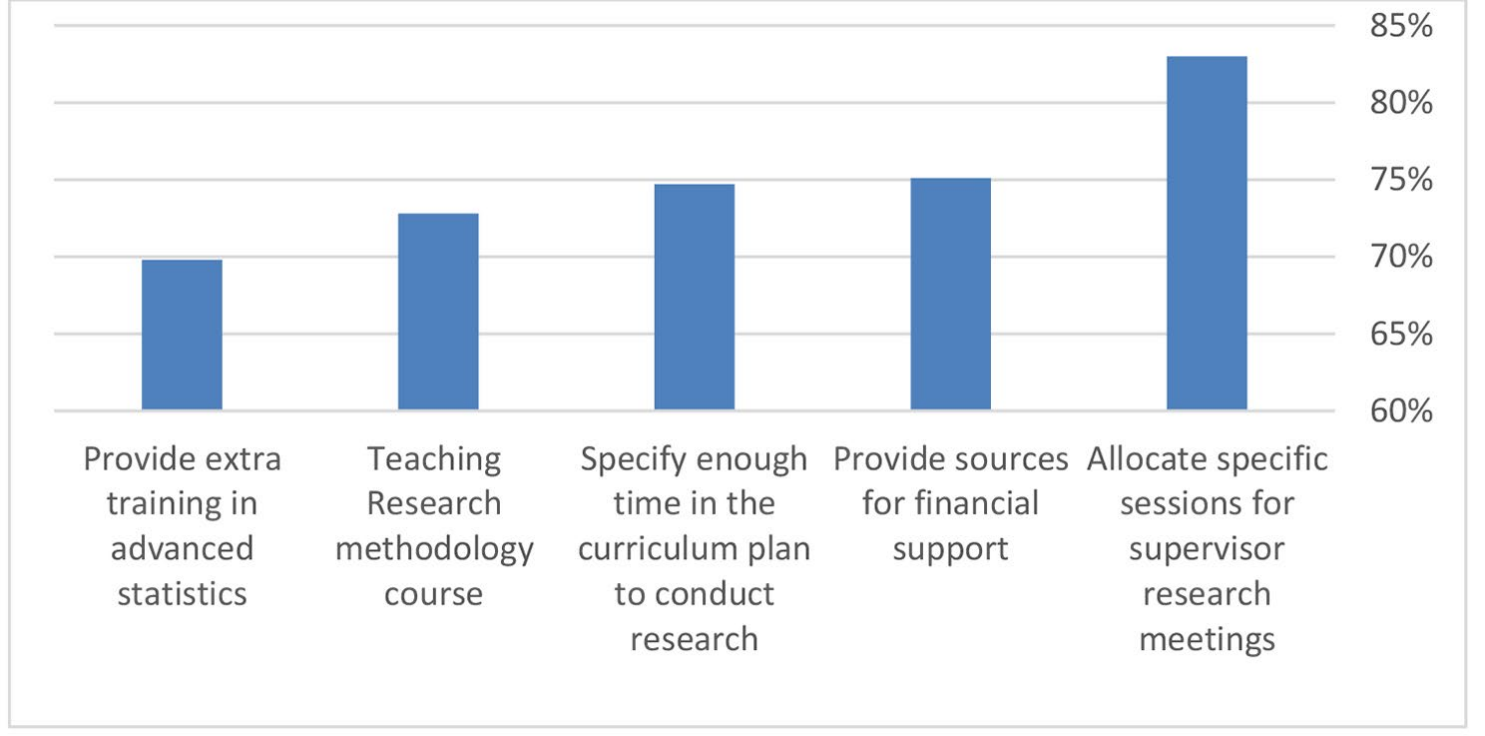
The student’s suggestions to improve engagement of undergraduates in research

84.9% of students suggested introducing special training modules for research supervisors. 76.5% recommended providing sources for financial support, and 76.2% suggested providing enough time in the curriculum plan to conduct research. 73.9% of students proposed research methodology courses, and 71.4% recommended additional training in advanced statistics. Further suggestions were, for example, to introduce a course on how to publish research work or to conduct research work primary after-graduation due to overload of curriculum Figure 4.

## Discussion

This study addresses medical student’s knowledge, attitude, and practices regarding research in medical colleges of Hadhramout University. Specifically, this study has embarked upon assessing the knowledge of medical students regarding research concepts and their attitudes toward research. Additionally, it aimed to describe the variations in research practices among medical students and to identify their perceived barriers to conducting research. Finally, the study sought out to suggest recommendations for the best research practices among medical students at Hadhramout University.

Our study suggests that knowledge about implementing research should be explored. We found that the majority of students understood the concept of research, sampling representation, and descriptive statistics. When comparing to the findings of other such researches, our finding was similar to research done by Louise N. Burgoyne et al. who found that the largest proportion of students in the study understood the term and concept of ‘research’ [20, 23]. However, we also found that students in our study couldn’t understand the concepts of inferential statistics, informed consent, or the need for sampling in methodology. Majumdar A et al. also found similar results, where knowledge in areas related to data entry and analysis software and scales of measurement performances in study designs and sampling techniques were also not satisfactory [24].

The result of this study revealed that the vast majority of medical students had a positive attitude toward research although; the majority of them had low knowledge scores. This finding is inconsistent with the findings of several studies [2, 23, 24]. Furthermore, a research which had been done previously showing that students with a prior degree or research experience have better attitudes towards research [19]. It is an advantage that students who had a high attitude toward science will tend to be highly involved in research activities, [25] and teaching research methodology improve the student’s attitude toward research [26]. Since conducting research is mandatory in Hadhramout University, such findings are plausible.

In our current study our findings also point out that female students had greater knowledge than males, whilst their attitudes to research did not differ. Similar results were obtained by Amin et al. [2] and Memarpouret [22]. In contrast, studies in Pakistan and USA revealed that male medical students showed a better attitude to research than female peers [18, 27–29]. The differences may be related to data collection from different populations, variations in sample size, [28] and the increasing acceptance of female students in our medical universities.

When addressing the research practice, we found more than three-quarters of participants reported that they had participated in research activities. This may be attributed to the mandatory research course done in the early years of scientific education in HUCOM, where a problem-based learning (PBL) approach is used, and also a similar policy appears to be implemented in many universities [2, 28]. Considerations of research in the curriculum varies across countries and universities [17, 30]. It was documented that involvement of the medical students in research is further reorganized in the curriculum of the PBL approach [25]. Vujaklija et al., Khan et al., and Wang and Guo stated that assessed projects and mandatory research improve experience and training in research. Moreover, a positive impact on students is reported, which motivates them to undertake further research in the future [26, 27, 31].

The barriers in conducting research which perceived by students were explored. Time was found to be the most significant barrier to pursuing research, followed by a lack of training statics, curriculum overload, lack of a competent supervisor, and poor access to the internet. Similar results had also been reported in other studies [2, 19, 22, 32]. Lack of time came up as an important barrier, as students were not interested in conducting research, and often identified time constraints as the reason [32]. Our finding revealed that 74.7% of students recommended specifying enough time in the curriculum for planning to conduct research.

The students pointed out several recommendations for improvement of undergraduate engagement in our research. The allocation of the specific session for research with supervisor meetings having the highest recommendation. Also, they recommended being provided with extra training in advanced statistics, and the provision of financial support. These recommendations were also found in several other studies [19–21].

### Limitation of the study

Limitation of this study was that the study targets the individual capacity of students while students in Hadhramout University conducted research in groups. This study emphasized the expected gap that the group work precludes the individual capacity.

### Study implications

the findings of this study may have three implications in the field of medical education. First, it develops the capacity of the undergraduate students to do research in their postgraduate period. Second, training undergraduate students about having research to develop their skills of data analysis, critical reading of the literature and scientific writing, and third; consequently, develop the ability of students to publish articles of better quality. To apply the above-mentioned activities, we advise the college to modify the curriculum and give enough time for students to accomplish their research without overload, and to provide to them competent supervisors.

## Conclusion

Knowledge of undergraduate medical students about research is low. Two thirds of students know the concept of research, sampling method and descriptive statistics. Most of them have low knowledge regarding sample size calculation and inferential statistics. Only one third of students were able to identify the correct definition of the informed consent. Although knowledge score of undergraduates was low, they however, expressed positive attitude toward research and this reflect their high interest but with low competency. Four main barriers to research were identified by students and should be taken in consideration: lack of sufficient time, lack of sufficient training on statistics, curriculum overload and lack of competent supervisors. Even with the above-mentioned barriers, the students research work has implication on their capacity of data analysis, critical reading of the literature and scientific writing.

## Data Availability

All data sets are available and can be shared by requesting it from the corresponding author by email.

## Abbreviations

ANOVA: Analysis Of Variance
CRF: Clinical Research Fellowship
HUCOM: Hadhramout University College Of medicine
NIH: National Institutes of Health
PBL: Problem Based Learning
SPSS: Statistical Package for Social Science
